# Comparative Evaluation of Cyclic Fatigue Resistance of EdgeTaper Platinum and ProTaper Gold Rotary Instruments: A Systematic Review and Meta-Analysis

**DOI:** 10.7759/cureus.108429

**Published:** 2026-05-07

**Authors:** Vivek Kalluri, Manoj Ramugade, Abrar Sayed, Kishor D Sapkale, Divya Nalavade, Prajakta Pol, Sanpreet S Sachdev

**Affiliations:** 1 Conservative Dentistry and Endodontics, Government Dental College and Hospital, Mumbai, IND; 2 Oral Pathology and Microbiology, Bharati Vidyapeeth (Deemed to Be University) Dental College and Hospital, Navi Mumbai, IND

**Keywords:** cyclic fatigue resistance, edgetaper platinum, meta-analysis, nickel-titanium rotary instruments, protaper gold, systematic review

## Abstract

Cyclic fatigue fracture remains a major concern during root canal instrumentation, particularly in curved canals. EdgeTaper Platinum and ProTaper Gold (PTG) are heat-treated nickel-titanium rotary systems designed to improve mechanical performance, but the available evidence comparing their cyclic fatigue resistance is inconsistent. This systematic review and meta-analysis was conducted to compare the cyclic fatigue resistance of these two systems. A systematic search was performed in PubMed, Scopus, Embase, EBSCOhost, and Google Scholar, along with manual searching and reference screening. No restrictions were applied for language or year of publication. In vitro studies comparing EdgeTaper Platinum and PTG for cyclic fatigue resistance were included. Data extraction and study selection were performed independently. Risk of bias was assessed using the QUIN (QUality assessment tool for IN vitro studies) tool. Quantitative synthesis was performed using a random-effects model. Seven in vitro studies were included in the qualitative synthesis, and four studies were included in the primary meta-analysis. Most individual studies reported higher cyclic fatigue resistance for EdgeTaper Platinum, although one study favored PTG and one did not show a clear direct pairwise difference. The pooled analysis demonstrated a statistically significant overall effect in favor of EdgeTaper Platinum for cyclic fatigue resistance. However, substantial heterogeneity was observed across studies, likely due to differences in canal geometry, file size, testing temperature, and environmental conditions. Within the limitations of the available in vitro evidence, EdgeTaper Platinum showed a more consistent advantage in cyclic fatigue resistance than PTG. Nevertheless, the high heterogeneity among studies suggests that this advantage is not uniform across all testing models, and more standardized comparative studies are needed.

## Introduction and background

Root canal treatment remains one of the most important procedures in conservative dentistry because its primary aim is to preserve teeth affected by pulpal and periapical disease [1,2]. A major step in successful endodontic therapy is biomechanical preparation of the root canal system, which should achieve adequate cleaning and shaping while maintaining the original canal path [1]. Successful preparation depends not only on effective debridement but also on the ability of the instrument to follow the natural canal anatomy with minimal procedural errors. Over the years, the shift from stainless steel hand instruments to nickel-titanium rotary systems has significantly improved endodontic practice [2,3]. Nickel-titanium instruments offer greater flexibility, better centering ability, and improved efficiency, especially in curved canals where conventional stainless steel files are more likely to cause procedural errors such as ledging, transportation, or zipping [3-5]. These advantages have made rotary instrumentation an integral part of contemporary endodontics [2,5].

Despite these advances, instrument separation remains a major concern during rotary root canal preparation [2,6]. Separation may interrupt cleaning and shaping procedures, complicate further instrumentation, and adversely affect treatment by limiting disinfection and obturation in the apical part of the canal [6]. Among the different modes of failure, cyclic fatigue is particularly important because it occurs when an instrument repeatedly bends and straightens while rotating in a curved canal, creating cycles of tension and compression within the metal [6,7]. Over time, these repeated stresses can initiate small cracks that gradually enlarge until fracture occurs [6,7]. Because this type of fracture may occur without visible warning, it is of particular clinical concern [6]. Cyclic fatigue resistance is influenced by several factors, including alloy composition, heat treatment, cross-sectional design, taper, canal curvature, radius, temperature, and the surrounding medium [7,8]. For this reason, newer rotary systems are continually being introduced with modifications in metallurgy and design to improve flexibility and fatigue resistance [8-10].

In recent years, increasing attention has been given to heat-treated nickel-titanium systems because thermal processing can modify the internal structure of the alloy and, in turn, influence flexibility, resistance to fracture, and overall mechanical behavior [8,11]. In simple terms, heat treatment can make an instrument behave in a more flexible manner under clinical conditions, which may help it better withstand repeated stress inside curved canals [8,11]. Among these systems, ProTaper Gold (PTG) and EdgeTaper Platinum (ETP) have attracted considerable attention because both are promoted as improved instruments with enhanced mechanical properties [11-13]. PTG uses proprietary gold heat treatment, whereas ETP has been associated with FireWire technology and design modifications intended to improve flexibility and resistance to fracture. Since both systems are used in similar clinical situations and are positioned as advanced heat-treated instruments, their comparative performance is of clear practical relevance to clinicians [11-13].

However, the available in vitro literature comparing ETP and PTG under controlled laboratory conditions remains limited, and the reported findings are not fully consistent. Some studies have favored one system over the other, while others have shown that the observed differences depend on testing conditions such as artificial canal design, curvature, testing medium, and temperature [13,14]. This variability makes direct interpretation difficult and limits the ability to draw a clear overall conclusion from individual studies alone [14]. Therefore, a critical appraisal and synthesis of the available evidence is required. The aim of the present review was to evaluate and compare the cyclic fatigue resistance of ETP and PTG rotary file systems, and the objective was to determine which system demonstrates superior performance under simulated clinical conditions.

## Review

Methodology

The present systematic review and meta-analysis were designed and reported in accordance with the Preferred Reporting Items for Systematic Reviews and Meta-Analyses (PRISMA) 2020 statement [15]. The review protocol was developed based on the PICO (Population, Intervention, Comparator, Outcome) framework to compare the cyclic fatigue resistance of ETP and PTG rotary file systems under simulated laboratory conditions. The protocol was registered in the PROSPERO database (Reference ID: CRD420251063005) before completion of article selection and the beginning of the data extraction process. The focused question of the review was: Which rotary file system, ETP or PTG, demonstrates better cyclic fatigue resistance?

Eligibility Criteria

The population comprised artificial root canals or standardized cyclic fatigue testing models. The intervention was PTG rotary instrumentation, and the comparator was ETP rotary instrumentation. The primary outcome was cyclic fatigue resistance, assessed mainly as the number of cycles to fracture or the time to fracture. Only in vitro experimental studies directly comparing these two systems were considered eligible. Studies were included if they used standardized cyclic fatigue models and reported relevant fatigue outcomes. Studies were excluded if they did not include both file systems, did not perform cyclic fatigue testing, used fractured or previously used instruments, were animal or clinical studies, or did not provide sufficient methodological standardization of the testing conditions.

*Search Strategy*

A comprehensive electronic literature search was performed in PubMed, Scopus, Embase, EBSCOhost, and Google Scholar. In addition, manual searches of major endodontic journals and cross-referencing of the reference lists of relevant studies were conducted to identify additional eligible reports. The search was updated through December 2025. No restrictions were applied for language or year of publication. Google Scholar results were sorted by relevance, and the first 200 records were screened for potential inclusion. The search strategy combined controlled vocabulary, where available, with free-text terms related to PTG, ETP, cyclic fatigue resistance, number of cycles to fracture, nickel-titanium rotary instruments, and in vitro testing. A representative electronic search string was as follows: ("ProTaper Gold" OR "Protaper Gold" OR "EdgeTaper Platinum" OR "EdgeTaper platinum") AND ("cyclic fatigue" OR "cyclic fatigue resistance" OR "number of cycles to fracture" OR NCF) AND ("nickel titanium" OR "NiTi" OR endodontic OR "rotary file" OR "rotary instrument") AND ("in vitro" OR laboratory OR experimental). The complete database-specific search strategies are provided in Appendix A.

Study Selection

Study selection was performed independently by two reviewers in two stages. In the first stage, titles and abstracts of all retrieved records were screened for relevance. In the second stage, the full texts of potentially eligible studies were assessed against the predefined inclusion and exclusion criteria. Disagreements between reviewers were resolved by discussion. When consensus could not be reached, a third reviewer was consulted for the final decision. Corresponding authors were contacted when clarification of study details was required.

Data Extraction

Data extraction was carried out independently by two reviewers using a pilot-tested, customized data extraction form. The following data were collected from each included study: author, year, country, study design, sample size, file system and specifications, canal model, testing conditions, outcome assessed, and main findings. Additional variables, such as curvature angle, curvature radius, testing medium, environmental temperature, and rotational settings, were also recorded when available because these factors were considered relevant to the interpretation of cyclic fatigue behavior.

Risk of Bias Assessment

Methodological quality was assessed using the QUality assessment tool for IN vitro studies (QUIN), which was specifically developed and validated for risk-of-bias assessment in dental in vitro research [16]. The QUIN tool evaluates 12 methodological domains, including aims and objectives, sample size calculation, sampling technique, comparison groups, methodological description, operator details, randomization, outcome measurement, assessor details, blinding, statistical analysis, and presentation of results. Based on the final score, studies were categorized as having low, medium, or high risk of bias. Because the included evidence was entirely in vitro, QUIN was considered more appropriate than tools intended for randomized clinical trials.

Certainty of Evidence

The certainty of evidence across the included studies was planned to be assessed using the Grading of Recommendations Assessment, Development, and Evaluation (GRADE) approach [17]. This framework considers the overall confidence in the body of evidence on the basis of study limitations, inconsistency, indirectness, imprecision, and publication bias. Since the included studies were laboratory-based and clinically indirect in nature, the certainty of evidence was expected to be interpreted cautiously.

Data Synthesis

A qualitative synthesis was first performed for all included studies. When sufficiently comparable quantitative data were available, meta-analysis was performed using Review Manager software (RevMan 5, Version 5.4. Copenhagen: The Cochrane Collaboration, 2020). The primary pooled outcome was cyclic fatigue resistance, expressed mainly as the number of cycles to fracture. Statistical heterogeneity among studies was assessed using the chi-square test and quantified with the I² statistic. A random-effects model was used when heterogeneity exceeded 50%, whereas a fixed-effect model was planned for lower heterogeneity. Effect estimates were calculated with 95% confidence intervals, and statistical significance was set at p<0.05. Because some studies reported multiple subgroup comparisons under different testing conditions, only one comparison arm per study was included in the primary meta-analysis to avoid duplication of data within the main pooled model. The remaining subgroup comparisons were considered in the qualitative synthesis.

Results

**Figure 1:**
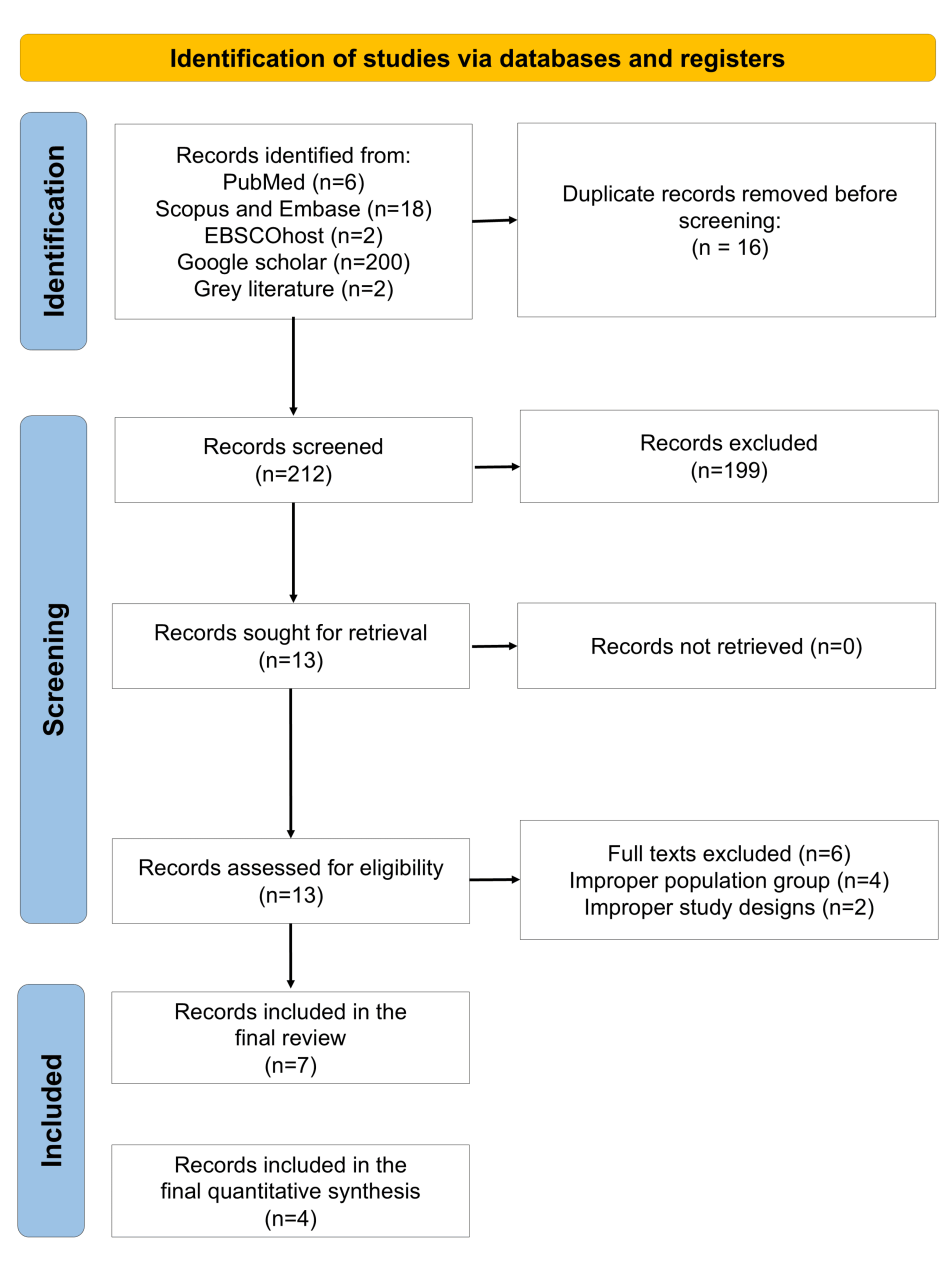
PRISMA flow diagram indicating the study selection process in the present systematic review PRISMA, Preferred Reporting Items for Systematic Reviews and Meta-Analyses

Out of the 228 identified search results, 212 records were screened after removal of duplicates. Of these, 13 full texts were retrieved. Ultimately, seven in vitro comparative studies fulfilled the eligibility criteria and were included in the final qualitative synthesis (Figure 1) [18-24]. Of these, four studies provided data suitable for quantitative synthesis, yielding six comparisons for meta-analysis. The characteristics of the included studies are summarized in Table 1.

**Table 1:** Characteristics of included studies comparing cyclic fatigue resistance of ETP and PTG ETP, EdgeTaper Platinum; FG, Flex Gold; NCF, number of cycles to failure; NR, not reported; PT, Pro-T; PTG, ProTaper Gold; EDTA, ethylenediaminetetraacetic acid; NaOCl, sodium hypochlorite; ISO, International Organization for Standardization

Study	Country	Study design	Files/specification	Sample size	Canal model	Testing conditions	Primary outcome	Main finding
Weyh and Ray, 2020 [18]	NR	In vitro comparative study	PTG vs. ETP; exact size NR	30 files of each type and size	3-point cyclic fatigue apparatus; 60° curvature; 3 mm radius	Other conditions NR	Cyclic fatigue resistance	ETP showed significantly greater cyclic fatigue resistance than PTG
Alcalde et al., 2020 [19]	Brazil	In vitro comparative experimental study	ETP 25/.06; PTG 25/.08; PT and FG also tested	n=30/system overall; cyclic fatigue subgroup n=10/system	Simulated artificial stainless-steel canal; 60° curvature; 5 mm radius; 0.40 mm apical diameter; 0.06 taper	35±1°C; deionized water bath; static test; ETP/PT/PTG at 300 rpm and FG at 500 rpm	Time to fracture; NCF	ETP showed higher cyclic fatigue resistance than PTG; PTG showed higher torsional strength
Zanza et al., 2022 [20]	Italy	In vitro comparative experimental study	PTG F2 vs. ETP F2; 25 mm	Total n=130; cyclic fatigue subgroup n=20/type	Tapered stainless-steel artificial canal; 16 mm length; 60° curvature; 5 mm radius	35±1°C saline bath; 300 rpm; torque limit 2.5 Ncm	Time to fracture; NCF	ETP showed higher cyclic fatigue resistance than PTG; PTG showed higher torsional resistance
Martins et al., 2022 [21]	Portugal/Brazil	In vitro multimethod experimental study	PTG F1 vs. ETP F1 within a 6-system comparison	n=50/group overall; n=12/group for each mechanical test	Stainless-steel custom tube; 86° curvature; 6 mm radius; 1.4 mm inner diameter	300 rpm; 2.0 N torque; glycerin lubricant; static test	Time to fracture	ETP showed greater flexibility than PTG; direct pairwise cyclic fatigue superiority between PTG and ETP was NR
Alfawaz et al., 2022 [22]	Saudi Arabia	In vitro comparative experimental study	PTG F2 vs. ETP F2; #25/.08; 25 mm	60 PTG + 60 ETP; n=20/group/solution	Stainless-steel artificial canal; 60° curvature; 5 mm radius; curvature center 5 mm from tip	37±1°C; 300 rpm; canals filled with 17% EDTA, 5.25% NaOCl, or distilled water	NCF	ETP showed higher cyclic fatigue resistance than PTG in all tested solutions; NaOCl reduced NCF in both systems
Hiran-us and Morakul, 2023 [23]	Thailand	In vitro comparative experimental study	PTG F5 vs. ETP F5; 50/.05 variable taper	20 files/system; 10 at room temperature and 10 at body temperature	Steel artificial canal; 60° curvature; 5 mm radius	Distilled water; 20±1°C and 37±1°C; 300 rpm; torque-control motor 3.1 N·cm	NCF	ETP showed higher cyclic fatigue resistance than PTG at both temperatures; NCF decreased at body temperature in all systems
Yum et al., 2024 [24]	Republic of Korea	In vitro experimental study	PTG F2 and ETP F2 included within a multigroup comparison; ISO #25; 25 mm	n=12/group	Artificial stainless-steel canal; 90° curvature; 2 mm inner radius and 3.5 mm outer radius	36±1°C water bath; 300 rpm; glycerin lubricant; static test	NCF	PTG showed higher cyclic fatigue resistance than ETP; ETP showed higher torsional resistance and stiffness

All included studies were laboratory-based investigations evaluating cyclic fatigue resistance in standardized artificial canal models. ETP and PTG were compared either directly or within multigroup mechanical assessments. Most studies used continuous rotation at 300 rpm, although instrument size, canal geometry, test medium, and environmental temperature varied across studies. Canal curvature ranged from 60° to 90°, and the radius of curvature ranged from 3 mm to 6 mm. The primary outcome was the number of cycles to failure (NCF) or time to fracture, while some studies also assessed torsional resistance, bending resistance, or related mechanical properties [18-24].

Qualitative Synthesis of Cyclic Fatigue Resistance

Five studies reported higher cyclic fatigue resistance for ETP than for PTG [18-20,22,23]. In Weyh and Ray [18], ETP showed significantly greater cyclic fatigue resistance than PTG. In Alcalde et al. [19], ETP showed a longer time to fracture and a higher NCF than PTG (n=10/system), with time to fracture values of 290.5 and 145.2 seconds and corresponding NCF values of 1453 and 732, respectively. In Zanza et al. [20], ETP again showed higher cyclic fatigue resistance than PTG (n=20/type), with mean NCF values of 1487.0±75.5 and 786.5±81.5, respectively. In Alfawaz et al. [22], ETP demonstrated higher NCF than PTG in all three testing solutions (n=20/group/solution). In Hiran-us and Morakul [23], ETP showed the highest NCF at both room temperature and body temperature (n=10/group/temperature), with corresponding values of 1811.0±580.73 and 1047.0±219.24, compared with 832.0±116.89 and 409.0±117.04 for PTG.

In contrast, Yum et al. [24] reported the highest NCF for PTG, while ETP ranked second in the same testing model (n=12/group). Martins et al. [21] evaluated the two systems within a six-system comparison (n=12/group for each mechanical test); however, the reported data did not demonstrate a clear direct superiority of one system over the other for cyclic fatigue resistance. Thus, on qualitative assessment, most included studies favored ETP, whereas one study favored PTG, and one study showed no clear direct difference between the two systems [18-24].

Influence of Testing Conditions

Test conditions influenced cyclic fatigue behavior. In Hiran-us and Morakul [23], both systems showed lower NCF values at body temperature than at room temperature (n=10/group/temperature). In Alfawaz et al. [22], sodium hypochlorite markedly reduced NCF in both groups, whereas EDTA did not significantly reduce fatigue resistance compared with distilled water (n=20/group/solution). Additional mechanical findings were also reported in some studies. Alcalde et al. [19] and Zanza et al. [20] found higher torsional strength for PTG, whereas Yum et al. [24] reported higher torsional resistance and stiffness for ETP.

Risk of Bias

Risk of bias assessment using the QUIN tool indicated that the overall methodological quality of the included in vitro studies was largely acceptable (Table 2). Five studies [19-21,23,24] were categorized as having low risk of bias, one study [22] showed medium risk of bias, and one study [18] showed high risk of bias. Across the included evidence, the domains most consistently reported were clearly stated aims and objectives, appropriate comparison groups, methodological description, outcome measurement, statistical analysis, and presentation of results. However, operator details and outcome assessor details were commonly not specified, and sample size calculation was not uniformly reported, which reduced methodological transparency in some studies [18,22]. Overall, the risk-of-bias findings suggest that the evidence base was predominantly composed of studies with low-to-moderate methodological concerns, although the presence of one high-risk study should be considered when interpreting the overall strength and consistency of the review findings.

**Table 2:** Risk-of-bias assessment of included in vitro studies using the QUIN tool QUIN, QUality assessment tool for IN vitro studies

Study	Aims/objectives	Sample size calculation	Comparison group	Methodology	Operator details	Outcome measurement	Outcome assessor details	Statistical analysis	Presentation of results	Total score	Overall risk
Weyh and Ray, 2020 [18]	2	0	2	1	0	1	0	1	1	8/18	High
Alcalde et al., 2020 [19]	2	2	2	2	0	2	0	2	2	14/18	Low
Zanza et al., 2022 [20]	2	2	2	2	1	2	0	2	2	15/18	Low
Martins et al., 2022 [21]	2	2	2	2	0	2	0	2	2	14/18	Low
Alfawaz et al., 2022 [22]	2	0	2	2	0	2	0	2	2	12/18	Medium
Hiran-us and Morakul, 2023 [23]	2	2	2	2	0	2	0	2	2	14/18	Low
Yum et al., 2024 [24]	2	2	2	2	0	2	0	2	2	14/18	Low

Certainty of Evidence

Risk of bias was not downgraded because most included studies were judged to have low risk of bias, with only one medium-risk and one high-risk study in the review. Inconsistency was downgraded because the direction of effect was not uniform across studies, and the pooled analysis showed substantial heterogeneity (I²=90%). Indirectness was downgraded by two levels because all included evidence was derived from in vitro models using artificial canals and simulated testing conditions rather than clinical use in patients. Imprecision was downgraded because the body of evidence was based on a limited number of laboratory studies with variation in file size, canal geometry, testing medium, and temperature, which reduced confidence in the stability and applicability of the estimated effect. Publication bias could not be confirmed and was therefore not formally downgraded.

The certainty of evidence for the primary outcome was judged to be very low (Table 3). Although most included studies showed acceptable methodological quality, the overall certainty was reduced by substantial inconsistency in the direction and magnitude of results across studies, as well as by the very high statistical heterogeneity observed in the meta-analysis. In addition, all available evidence was derived from in vitro experimental models, which limits direct clinical applicability and represents serious indirectness. The small number of included studies and the variability in testing conditions, including canal curvature, radius, temperature, irrigating medium, and file specifications, also contributed to reduced confidence in the estimate. Therefore, the current evidence does not allow a high-certainty conclusion regarding the comparative cyclic fatigue superiority of ETP or PTG under clinical conditions.

**Table 3:** GRADE certainty of evidence for the primary outcome PTG, ProTaper Gold; ETP, EdgeTaper Platinum; GRADE, Grading of Recommendations Assessment, Development, and Evaluation

Outcome	No. of studies	Study design	Risk of bias	Inconsistency	Indirectness	Imprecision	Publication bias	Overall certainty	Interpretation
Cyclic fatigue resistance of ETP vs. PTG	7 studies (4 studies, 6 comparisons in meta-analysis)	In vitro comparative studies	Not serious	Serious	Very serious	Serious	Undetected	Very low	The true comparative effect of ETP vs. PTG on cyclic fatigue resistance remains very uncertain

Quantitative Synthesis

**Figure 2:**
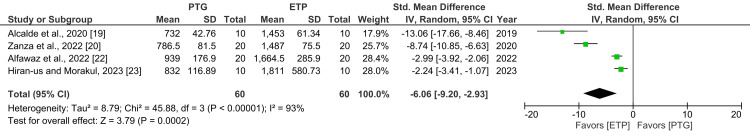
Forest plot showing pooled results in favor of ETP PTG, ProTaper Gold; ETP, EdgeTaper Platinum Source: [19,20,22,23]

Four studies were included in the meta-analysis, contributing one comparison each and a total of 60 instruments in the PTG group and 60 instruments in the ETP group [19,20,22,23]. Because between-study heterogeneity was substantial, a random-effects model was used. The pooled analysis showed a statistically significant overall effect in favor of ETP, with a standardized mean difference of -6.06 (p=0.0002). All included studies showed effect estimates on the side favoring ETP, although the magnitude of effect varied across studies [19,20,22,23]. Considerable heterogeneity was observed in the pooled analysis (p<0.00001; I²=93%), indicating marked variation in effect size across studies (Figure 2).

**Figure 3:**
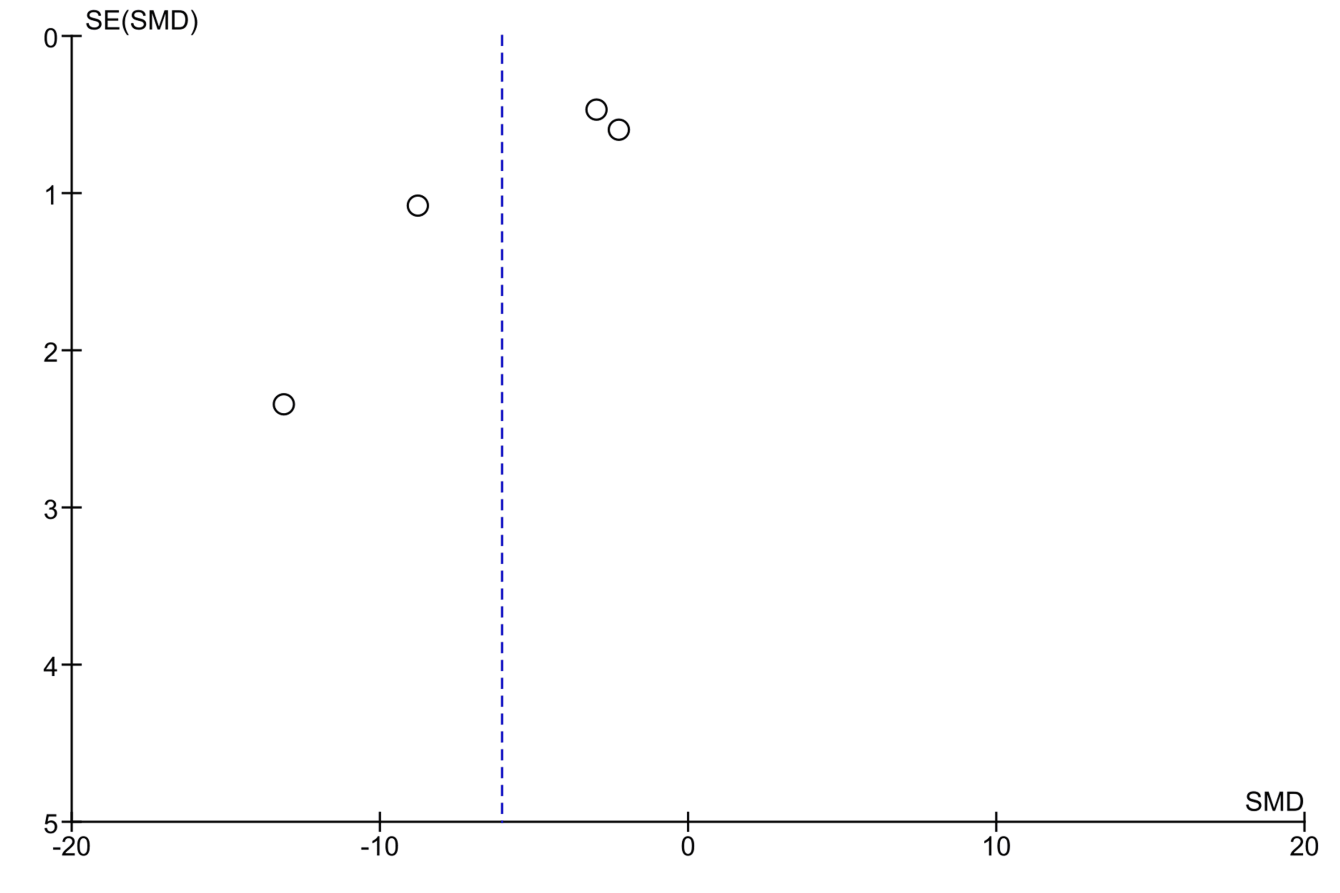
Funnel plot showing the presence of minor asymmetry The slightly scattered, asymmetrical distribution of the studies considered for meta-analysis indicates the presence of minor publication bias [19,20,22,23]. SE, standard error; SMD, standardized mean difference

Visual inspection of the funnel plot suggested asymmetry (Figure 3), with the included comparisons not distributed evenly around the pooled effect size. Although this may indicate possible small-study effects or publication bias, the limited number of comparisons in the meta-analysis restricts the reliability of funnel plot interpretation. Therefore, publication bias could not be confirmed with confidence.

Discussion

The present systematic review showed that the comparative cyclic fatigue performance of ETP and PTG was not entirely uniform across all experimental models. On qualitative synthesis, most included studies favored ETP, whereas one study favored PTG, and one study did not demonstrate a clear direct pairwise difference between the two systems [18-24]. The primary meta-analysis also showed a statistically significant pooled effect in favor of ETP, although the heterogeneity was very high. Taken together, these findings suggest that ETP demonstrated a more consistent advantage in cyclic fatigue resistance under the selected test conditions but that the size of this advantage varied substantially across studies.

Metallurgical and Design-Related Considerations

The differences observed between ETP and PTG cannot be explained by metallurgy alone. Heat treatment modifies the internal structure of nickel-titanium alloys and can influence flexibility, fatigue resistance, and fracture behavior [8,25]. This is one of the main reasons why heat-treated systems have attracted attention in contemporary endodontics. In the present review, ETP showed superior cyclic fatigue resistance in several direct comparisons [18-20,22,23], whereas PTG demonstrated the highest NCF in one multigroup study [24]. This inconsistency suggests that heat treatment is only one part of the explanation.

Instrument design is also likely to play an important role. The included studies did not all evaluate the same file size or taper, and in at least one study, the taper of ETP and PTG was not identical [19-24]. Variations in taper, core mass, and cross-sectional area can alter flexibility and internal stress distribution, thereby affecting cyclic fatigue resistance independently of the alloy treatment. This may explain why ETP frequently showed higher cyclic fatigue resistance or greater flexibility, whereas PTG showed better torsional strength in some comparisons [19,20,24]. Therefore, the current findings suggest that the mechanical performance of these systems is determined by the combined effect of heat treatment, geometry, and testing conditions rather than by any single factor alone.

Influence of Testing Conditions

One of the most important findings of this review was the marked influence of testing conditions on cyclic fatigue behavior. The included studies differed in canal curvature, radius, file size, taper, environmental temperature, and testing medium, all of which are known to affect cyclic fatigue resistance [7]. The angle of curvature ranged from 60° to 90°, while the radius ranged from 3 mm to 6 mm, indicating that the instruments were tested under varying levels of mechanical stress. These methodological differences are likely to have contributed to the considerable heterogeneity observed in the pooled analysis.

Temperature was a particularly important variable. Hiran-us and Morakul [23] showed that both ETP and PTG exhibited lower NCF values at body temperature than at room temperature, indicating that room-temperature models may overestimate fatigue resistance. This observation is supported by previous studies showing that temperature can alter the mechanical behavior of heat-treated nickel-titanium instruments through changes in phase transformation behavior [26,27]. Similarly, Alfawaz et al. [22] demonstrated that sodium hypochlorite reduced cyclic fatigue resistance in both systems, whereas EDTA did not significantly reduce fatigue resistance compared with distilled water. These findings are clinically relevant because rotary instruments function in a warm intracanal environment and are routinely exposed to irrigants during instrumentation [28].

Interpretation of the Pooled Findings

Although most individual studies favored ETP, the pooled estimate must be interpreted with caution because heterogeneity was substantial. The inconsistency in effect size across studies most likely reflects differences in canal configuration, instrument specification, and environmental conditions rather than a contradiction in the basic direction of findings. In the primary pooled analysis, all included comparisons were on the side favoring ETP, but the magnitude of the standardized effect varied markedly. This indicates that the comparative advantage of ETP may be present across several experimental conditions but is not likely to be uniform under all laboratory models.

The certainty of evidence for the primary outcome was judged to be very low. This rating was mainly due to serious inconsistency, very serious indirectness, and serious imprecision. Therefore, while the pooled data support a comparative advantage for ETP in cyclic fatigue resistance, the current evidence does not justify a strong conclusion regarding clinical superiority.

Limitations 

The limitations of the included evidence should be acknowledged. First, all included studies were in vitro investigations using artificial canal models, and none evaluated clinical outcomes in patients. Second, the studies varied substantially in canal design, curvature, radius, file size, taper, temperature, irrigating medium, and fatigue model. This reduced comparability across studies and likely contributed to the high heterogeneity of the pooled analysis. Third, although most studies were judged to have low risk of bias, reporting deficiencies were common, particularly with respect to operator details and outcome assessor details, and one study had a high risk of bias [18-24].

The review itself also has limitations. Quantitative synthesis was possible only for a subset of studies, and the pooled estimate was based on a small number of comparisons. In addition, one included report provided limited methodological detail, and some multigroup studies did not present a clearly stated direct pairwise comparison between ETP and PTG for cyclic fatigue. Funnel plot asymmetry was observed, but publication bias could not be confirmed because of the limited number of studies available for meta-analysis. These issues reduce confidence in the precision and generalizability of the summary effect.

Future Scope

Future research should focus on better standardization of cyclic fatigue testing protocols, including canal curvature, radius, temperature, rotational settings, and reporting of exact file dimensions and metallurgy. Comparative studies performed under clinically relevant body-temperature conditions and commonly used irrigating media would improve the practical value of laboratory findings. Direct head-to-head studies using equivalent file sizes and tapers are especially needed to reduce design-related confounding. More importantly, future work should determine whether the laboratory differences observed between ETP and PTG translate into clinically meaningful differences during root canal preparation. Until such evidence becomes available, the findings of the present review should be interpreted as comparative laboratory evidence rather than definitive proof of clinical superiority of one system over the other.

## Conclusions

Based on the included in vitro evidence, ETP demonstrated higher cyclic fatigue resistance than PTG in most direct laboratory comparisons, although this finding was not uniform across all studies. The pooled meta-analysis also showed an overall significant effect in favor of ETP, but the high heterogeneity indicates that the magnitude of this advantage varied considerably across testing models. Therefore, within the limitations of the available laboratory evidence, ETP appears to have a more consistent advantage in cyclic fatigue resistance, while further standardized and clinically relevant comparative studies are needed before drawing stronger clinical inferences.

## Appendices

Appendix A 

**Table 4:** Database-specific search strategies Searches were updated through December 2025. No restrictions were applied for language or year of publication. Google Scholar results were sorted by relevance, and the first 200 records were screened.

Database	Search strategy
PubMed	(“ProTaper Gold”[Title/Abstract] OR “Protaper Gold”[Title/Abstract] OR “EdgeTaper Platinum”[Title/Abstract] OR “EdgeTaper platinum”[Title/Abstract]) AND (“cyclic fatigue”[Title/Abstract] OR “cyclic fatigue resistance”[Title/Abstract] OR “number of cycles to fracture”[Title/Abstract] OR NCF[Title/Abstract]) AND (“nickel titanium”[Title/Abstract] OR NiTi[Title/Abstract] OR endodontic[Title/Abstract] OR “rotary file”[Title/Abstract] OR “rotary instrument”[Title/Abstract]) AND (“in vitro”[Title/Abstract] OR laboratory[Title/Abstract] OR experimental[Title/Abstract])
Scopus	TITLE-ABS-KEY (“ProTaper Gold” OR “Protaper Gold” OR “EdgeTaper Platinum” OR “EdgeTaper platinum”) AND TITLE-ABS-KEY (“cyclic fatigue” OR “cyclic fatigue resistance” OR “number of cycles to fracture” OR NCF) AND TITLE-ABS-KEY (“nickel titanium” OR NiTi OR endodontic OR “rotary file” OR “rotary instrument”) AND TITLE-ABS-KEY (“in vitro” OR laboratory OR experimental)
Embase	(‘protaper gold’:ti,ab,kw OR ‘edgetaper platinum’:ti,ab,kw) AND (‘cyclic fatigue’:ti,ab,kw OR ‘cyclic fatigue resistance’:ti,ab,kw OR ‘number of cycles to fracture’:ti,ab,kw OR ncf:ti,ab,kw) AND (‘nickel titanium’:ti,ab,kw OR niti:ti,ab,kw OR endodontic:ti,ab,kw OR ‘rotary file’:ti,ab,kw OR ‘rotary instrument’:ti,ab,kw) AND (‘in vitro study’:ti,ab,kw OR laboratory:ti,ab,kw OR experimental:ti,ab,kw)
EBSCOhost	TX (“ProTaper Gold” OR “Protaper Gold” OR “EdgeTaper Platinum” OR “EdgeTaper platinum”) AND TX (“cyclic fatigue” OR “cyclic fatigue resistance” OR “number of cycles to fracture” OR NCF) AND TX (“nickel titanium” OR NiTi OR endodontic OR “rotary file” OR “rotary instrument”) AND TX (“in vitro” OR laboratory OR experimental)
Google Scholar	“ProTaper Gold” OR “Protaper Gold” OR “EdgeTaper Platinum” OR “EdgeTaper platinum” AND “cyclic fatigue resistance” OR “number of cycles to fracture” OR NCF AND endodontic AND “in vitro”
Manual search	Manual searching of major endodontic journals and cross-referencing of relevant studies were also performed. Journals screened included International Endodontic Journal, Journal of Endodontics, Saudi Endodontic Journal, Journal of Conservative Dentistry, Australian Endodontic Journal, European Endodontic Journal, and British Dental Journal.
